# Light-stimulated micromotor swarms in an electric field with accurate spatial, temporal, and mode control

**DOI:** 10.1126/sciadv.adi9932

**Published:** 2023-10-25

**Authors:** Zexi Liang, Hyungmok Joh, Bin Lian, Donglei Emma Fan

**Affiliations:** ^1^ Materials Science and Engineering Program, Texas Materials Institute, The University of Texas at Austin, Austin, TX 78712, USA.; ^2^ Department of Mechanical Engineering, The University of Texas at Austin, Austin, TX 78712, USA.

## Abstract

Swarming, a phenomenon widely present in nature, is a hallmark of nonequilibrium living systems that harness external energy into collective locomotion. The creation and study of manmade swarms may provide insights into their biological counterparts and shed light to the rules of life. Here, we propose an innovative mechanism for rationally creating multimodal swarms with unprecedented spatial, temporal, and mode control. The research is realized in a system made of optoelectric semiconductor nanorods that can rapidly morph into three distinct modes, i.e., network formation, collectively enhanced rotation, and droplet-like clustering, pattern, and switch in-between under light stimulation in an electric field. Theoretical analysis and semiquantitative modeling well explain the observation by understanding the competition between two countering effects: the electrostatic assembly for orderliness and electrospinning-induced disassembly for disorderliness. This work could inspire the rational creation of new classes of reconfigurable swarms for both fundamental research and emerging applications.

## Teaser

An innovative optoelectric mechanism for creating multimodal swarms with spatial, temporal, and mode control is presented.

## INTRODUCTION

Swarming is a hallmark of nonequilibrium systems that can convert external energy into collective locomotion and patterning. The phenomenon is ubiquitously present in both live and inanimate systems made of constitutional units with sizes ranging from nanometers to meters (*1*). Some examples include molecular motor proteins (*2*), bacterial colonies, flocks of sheep, and swarms of drones. Recently, various artificial micro/nanoscale machines have been explored in creating swarms for both scientific investigation and applications. It is because, compared to their biological counterparts, artificial micromachines are structurally simpler, are size controllable, and can be propelled by diverse energy sources, including acoustic (*3*, *4*), optical (*5*–*8*), electrostatic (*9*), magnetic (*10*–*14*), and hydrodynamic fields (*15*) and chemical reactions (*6*–*8*, *16*–*23*). A swarm made of artificial micromachines also often offers intriguing properties not exhibited by its constituting individual units. For instance, optical stimulation of a collection of titanium dioxide (TiO_2_) micromotors can dramatically enhance the suspension’s viscosity by an order of magnitude (*6*, *24*); they can also form unique self-assembly (*12*, *25*) and exhibit long-range inter-droplet communication (*26*). In addition, intriguing applications of micromachine swarms have been demonstrated, ranging from picking up and dropping cargo (*8*), propelling through biological barriers (*13*, *27*, *28*), enhanced drug delivery and surgical operations (*13*, *29*), to water treatment (*30*). It is highly desirable to investigate a rational scheme for creating and manipulating micromachine swarms. The achievement of such could assist in understanding the rules of complex live systems and exploring emergent applications.

Extensive experimental and theoretical research has revealed the critical role of near-field interactions among the constituting units for creating swarming behaviors, including assembly and collective locomotion (table S1). In particular, magnetic interactions and hydrodynamics generate multiple swarming behaviors (*14*, *31*, *32*). In this work, we propose an innovative working mechanism by exploring light–semiconductor–electric field interactions to enable reconfigurable multimodal swarming. Such a swarming system can form versatile patterns and change its operation mode in response to simple projected light in an electric field. The method is via actively introducing two competing actuation effects with one generating near-field attraction and assembly (orderliness), and the other introduces disruptions of assembly (disorderliness); the relative weight is tunable from zero to one. The proposed scheme is inspired by the temperature-controlled thermodynamic phase transition in a binary alloy, where atomic bonding introduces assembly (orderliness) and thermal energy induces atomic mixing (disorderliness). The relative weight of these two competing factors controls phase transformation. Experimentally, we realized such a designed multimodal swarm system by exploring asynchronous rotation of photoconductive semiconductor Si rod micromotors in water in a high-frequency electric field that provides two countering electric effects, i.e., (i) assembling of neighboring motors due to near-field electrostatic force and (ii) disassembling of motors due to mechanical electrorotation. As a result, by controlling the weighted roles using light and AC frequency, we create three distinct swarming modes in a Si micromotor suspension, including two-dimensional (2D) networks, droplet-like clusters, and enhanced collective rotation. The modes all instantly interswitch and exhibit versatile spatial and time control. Theoretical analysis and semiquantitative numerical simulation well reproduce the dynamic features of all swarming modes. The results support the feasibility of the rational scheme of multimodal swarms.

## RESULTS

**Fig. 1.**
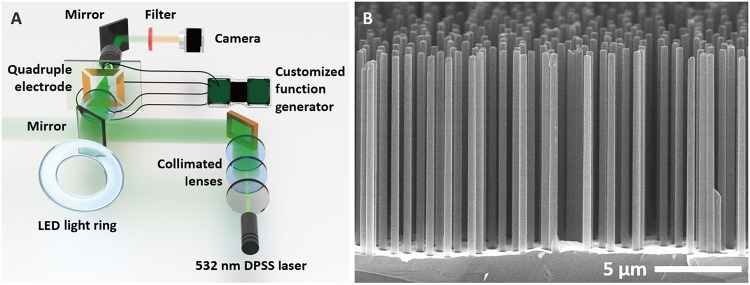
Electric field–driven optically modulated Si micromotor swarms. (**A**) Schematic of experiment setup. (**B**) Scanning electron microscopy images of silicon nanorods.

The optoelectric semiconductor silicon nanorod motors are fabricated by metal-assisted chemical etching (MACE), as discussed in detail in Methods. The nanorods are 500 nm in diameter and 7 μm in average length (up to ~10 μm) dispersed in solution; they are made from intrinsic single-crystal silicon wafers (Fig. 1A). A droplet of nanorods in deionized (DI) water is dispersed in the square region (500 μm × 500 μm) of a quadruple microelectrode. A high-frequency rotating AC field is created to manipulate the Si nanorods by applying four AC voltages [10 kHz to 1 MHz and up to 30 peak-to-peak voltage (*V*_pp_)] with sequential 90° phase shifts to the microelectrodes. The entire microchip is loaded on an inverted microscope (Olympus IX 71) equipped with a 532-nm laser (100 mW/cm^2^) and a digital light projector (D4100) (Fig. 1B).

Under light pattern illumination, the nanorods in solution demonstrate collective synchronous motion with near-field interactions. Three swarming modes are observed from 10 to 500 kHz, including light pattern–defined enhanced rotation (500 kHz) (movie S1), network formation (100 to 200 kHz) (movie S2), and droplet-like clustering (10 kHz) (movie S3). The three modes can be rapidly switched in between (movie S4); the shape, size, and location are also versatilely directed by light patterns.

### Electrorotation and the driving torque for disassembling

A rotating electric field applied to a Si rod is given by $E=E_{0}(\hat{x}-i\hat{y})\exp(i\text{ω}t)$, where the *x* axis along the nanorod's longitudinal direction and ω, *t*, and *E*_0_ are the frequency, time, and magnitude of the rotating electric field, respectively. The induced dipole **p** from an individual nanorod can be calculated by **p =** α**E_∥_**, where α is the electric polarizability. Then, the averaged electrorotation torque over one cycle can be expressed as follows (note S1)

τe=12Re[p(t)×E∗(t)]=−12E02Im[α]z^ (1)

where the underlines of **p***(t*) and **E***(t)* denote complex variables with a phasor, and the start symbol denotes complex conjugate. As shown in Eq. 1, the electrorotation, which introduces disassembly and disorderliness to Si micromotors, is governed by the imaginary part of electric polarizability, a factor tunable by the light-controlled electric conductivity of Si as well as the electric field frequency.

### Near-field electrostatic interaction for assembling

**Fig. 2.**
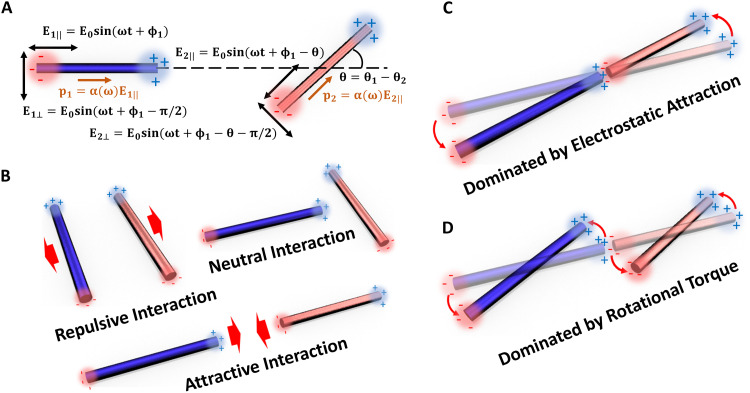
Induced dipole interaction between two micromotors. (**A**) Induced dipoles of micromotors in an arbitrary orientation under a rotating electric field. (**B**) Three representative configurations of micromotors with attractive/repulsive/neutral interactions. Two micromotors (**C**) assemble during rotation when electrostatic attraction dominates and (**D**) spin individually when rotational torque dominates.

For rod motors placed close by, a simplified point-charge model is adopted to quantitatively analyze the near-field electrostatic interaction (note S2), where an electric dipole induced on a Si motor is treated as a pair of point charges located at the two ends of a rod-shaped motor. This results in electrostatic interactions between two such motors placed next to each other (Fig. 2A). To facilitate the calculation, the center positions, orientations, and the unit vector along the long axes of the two micromotors are defined as **r**_1_, θ_1_, ${\hat{s}}_{1}$, and **r**_2_, θ_2_, and ${\hat{s}}_{2}$, respectively. The dipole-dipole Coulomb interaction between rods 1 and 2 is then calculated from the four point charges *q*_1_^+^, *q*_2_^+^, *q*_1_^−^, and *q*_2_^−^. With careful calculation and analysis shown in note S2, we obtain the average Coulomb force between rods 1 and 2 during one cycle of electric field rotation in Eq. 2

$$F_{12}=\frac{\cos(\theta_{2}-\theta_{1})}{2}\frac{1}{4\pi \varepsilon_{m}}\frac{q_{0}^{2}}{{|r_{1}^{\pm }-r_{2}^{\pm }|}^{2}}$$ (2)

where $q_{0}=\frac{\alpha E_{0}}{l}$ is the induced charge on a rod motor when it aligns with the external field **E**, ɛ*_m_* is the electric permittivity of suspension medium, and *F*_12_ is the sum of the mutual interactions of all the four point charges. Equation 2 shows that the electrostatic interaction is proportional to the electric polarization and depends on the angular configurations of the two micromotors. For instance, the force is zero when two rod motors are orthogonal to each other.

Note that the model provides understanding of the experimental observations with the following approximations and assumptions: (i) The induced dipoles on rods are approximated as point charges; (ii) all rod motors are assumed to be identical in geometry and property; (iii) the dipole moment of an individual rod motor solely depends on the external field, and the induced field from a neighboring motor is omitted; (iv) only the polarization along the long axis is considered; and (v) the hydrodynamic interactions between different rod motors are omitted since electrostatic forces dominate over hydrodynamic forces in most experimental conditions unless noted otherwise. The above assumptions and approximations can provide reasonable understanding in terms of a semiquantitative modeling for the comparison with experiments.

Equation 2 readily sheds light to near-field dipole-dipole interactions among the rod micromotors. We consider several simple configurations and interactions between two identical rods in an electric field. As shown in Fig. 2B, owing to synchronously created dipoles of the same polarity, attraction occurs when two rods are placed head-to-tail (θ_1_ = θ_2_), repulsion presents when arranged shoulder-to-shoulder (θ_1_ = θ_2_ + π), and the interaction becomes zero when placed orthogonally ($\theta_{1}=\theta_{2}+\frac{\pi }{2}$). When both rods explore all possible angles, the total interactive force is attractive. This is shown by the simulation in detail in note S3. The strength of the interaction increases with the square of the dipole moment (*p*^2^) and electric field (*E*^2^).

We also note that when two neighboring rod motors are in the head-to-tail arrangement, the force from the dipole-dipole interaction favors tip-to-tip assembly. However, the electrostatic attraction may be overcome when the electrorotation torque from the dipole-field interaction is sufficiently strong, leading to independent spinning of the two micromotors. As a result, the two motors can form an assembly and rotate as a single entity when the attractive force dominates (Fig. 2C) and spin along their geometrical center as individuals when the rotational torque dominates (Fig. 2D). The competition between these two forms of actuation establishes the foundation of achieving swarming and their reconfigurable mode switching. The relative weight of the two countering effects can be tuned by controlling light illumination and electric fields applied to the Si rod motors.

### Understanding of competing interactions between a pair of neighboring motors

The competing effects on a Si motor discussed above and their relative weight can be quantitatively understood by theoretical calculations. In Eqs. 1 and 2, Im(**p**) and the total electric polarization **p** govern the electrorotation torque and the dipole-dipole interaction between two neighboring rod motors, respectively. Expanding on Eqs. 1 and 2, we calculate Im(**p**) and the total electric polarization **p** of Si rod motors in DI water, considering both the Maxwell-Wagner electric polarization of Si and electric double-layer effect in water in an electric field (*33*, *34*).

**Fig. 3.**
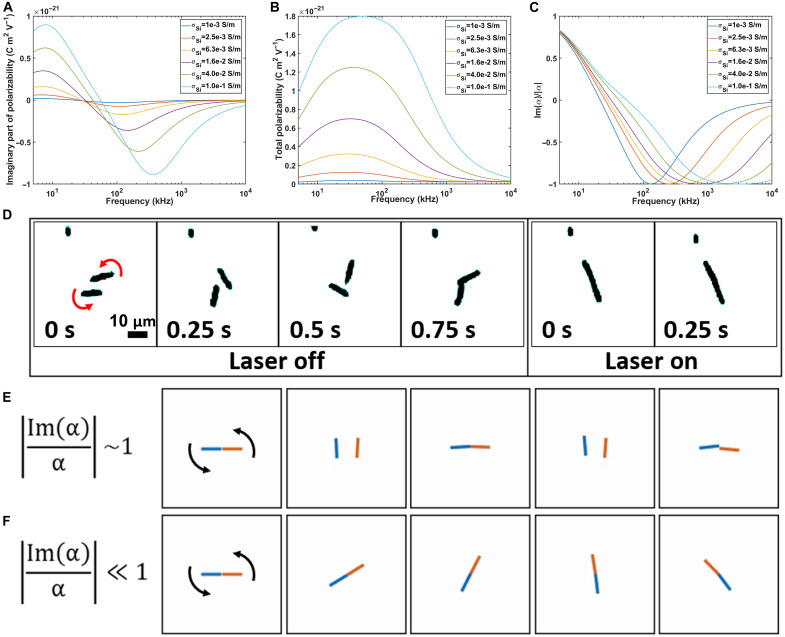
Simulation of interaction dynamics of two neighboring Si rod micromotors and experimental validation. (**A** to **C**) Calculation of (A) the imaginary part of polarization, (B) the total polarization, and (C) their ratio as a function of frequency and electric conductivity of a Si rod micromotor. (**D**) Interactions of two rods rotating at 50 kHz (60,000 V/m) with and without 532-nm laser illumination (100 mW/cm^2^). (**E** and **F**) Brownian dynamic simulation of two motors rotating when |Im(α)/α| approaches (E) one and (F) zero, respectively.

We first determine the imaginary part of the dipole moment [Im(**p******) of a silicon motor (500 nm in diameter and 7.5 μm in length] under illumination in DI water and in an electric field of 60,000 V/cm (Fig. 3A). When Im(**p**) is positive, the rod motor spins opposite to the field rotation, and vice versa. The Im(**p**) exhibits positive, zero, and negative values depending on the frequency. At around 10 kHz, its magnitude reaches the positive maximum and gradually decreases to the negative maximum in a frequency range of 100 to 500 kHz depending on the electric conductivity of the photoconductive Si rods, which can be easily modulated by light. Without laser exposure, the electric conductivity is estimated to be around 10^−3^ S/m, on the same level of that of intrinsic silicon at room temperature, and the corresponding Im(**p**) is minimum (Fig. 3A, dark blue curve) (*33*). When exposed to a 532-nm laser at 100 mW/cm^2^, the electric conductivity increases to ~10^−1^ S/m (*33*), and Im(**p**) of the Si micromotor increases substantially in magnitude across the overall frequencies (Fig. 3A, light blue curve).

Different from electrorotation, the electrostatic interaction governs the near-field assembly of nanorods and is determined by the total electric polarization **p** (Fig. 3B); this effect also strongly depends on the electric field frequency but in a distinct fashion compared to that of electrorotation in Fig. 3A. The total electric polarization **p** increases with AC frequency and reaches a plateau before it gradually decreases to near zero; the magnitude monotonically increases with the light-modulated electric conductivity across the entire frequency range and reaches its highest value at 20 to 100 kHz, tuned by the electric conductivity. Overall, for Si rod motors, both Im(**p**) and total electric polarization **p** demonstrate strong, but distinct, frequency dependence. The magnitudes of both effects increase with the intensity of light illumination.

The above calculations and analysis readily guide our experimental studies on a pair of close-by Si micromotors. For instance, Fig. 3 (A and B) indicates that at 100 kHz and under light illumination, the electrorotation torque on a Si rod is low but the total electric polarization (**p**) is close to the highest value. Therefore, at 100 kHz, according to the theoretical calculation, electro-attraction dominates the two rods’ interaction. Experimentally, when we apply an electric voltage at 100 kHz and gradually increase it from 0 to 10 V, two neighboring Si nanorods interact and finally attract and assemble tip-to-tip; almost no rotation is observed during the voltage ramping (movie S5). The experiments agree with the theoretical predictions.

To provide an easy and direct guidance to the experimental study, we further calculate Im(α)∣α∣ versus AC frequency (Fig. 3C); here, the sign of Im(α)∣α∣ only indicates two different rotation directions. The absolute value ∣Im(α)α∣ varies from 0 to 1 and directly reflects the relative weight of the two countering effects. When approaching 1 and 0, the rotation torque and electrostatic attraction dominates, respectively.

Such two extremes, indicated by the theoretical study, can be realized at 50 kHz on two neighboring Si rods with controlled laser illumination. Experiments at 50 kHz show that two neighboring Si rod motors spin independently and rapidly assemble tip-to-tip, with and without light, corresponding to ∣Im(α)α∣→1and→0, respectively. The light-controlled switch is instantaneous and reversible (Fig. 3D and movie S6).

Moreover, the experimental observations are well reproduced by our dynamic simulations; when ∣Im(α)α∣→1, the two simulated neighboring rods spin independently (Fig. 3E and movie S7), while, when ∣Im(α)α∣≪1, the simulated rod motors assemble and rotate as single entities (Fig. 3F and movie S8); the results correspond to the dominating roles of rotational torque and near-field electrostatic interaction, respectively. The agreement of the experimental results and theoretical studies on a pair of Si motors validates our understanding of the interactions between close-by motors. There can be a variance in the length of the fabricated rods, leading to different Im(**p**) and (**p**) values under light. Measuring ~100 randomly chosen nanorods under two separate experiments shows that the rods are ~7 μm long on average following a normal distribution (fig. S1). Nevertheless, additional simulations of 5- and 10-μm wires indicate that while the Im(**p**) and (**p**) values can slightly shift with the length, the ∣Im(α)α∣ value is similar for all cases (fig. S2), leading to similar responses at the same conditions.

### Multimodal reconfigurable micromotor swarms: Experiments and simulation

For a low-density Si motor suspension, the interaction among the neighboring motors is too low to generate any collective motion due to the large separation distance among the motors. The induced dipole-dipole interaction between neighboring motors becomes prominent with the increase of the motor density and corresponding reduced inter-distance. In particular, in addition to the dipole-field interaction, the dipole-dipole interaction between photoconductive Si motor pairs can be readily tuned by light in an electric field. As a result, in an electric field with a strength that the dipole-dipole interactions are insufficient for supporting a collective motion, the interactions can become much stronger under light, leading to collective swarming locally in the light-illuminated region. Guided by these understandings, we demonstrate three interswitchable swarming modes with high-density Si motor suspension by controlling the light exposure (532-nm laser, 100 mW/cm^2^) and electric field (10 kHz to 1 MHz, 60,000 V/m). The swarms instantly appear into patterns in light-defined areas, rapidly reconfigure into a different swarming mode, and vanish on demand. We also provide a qualitative understanding of the observations using a simple electrostatic-based model.

**Fig. 4.**
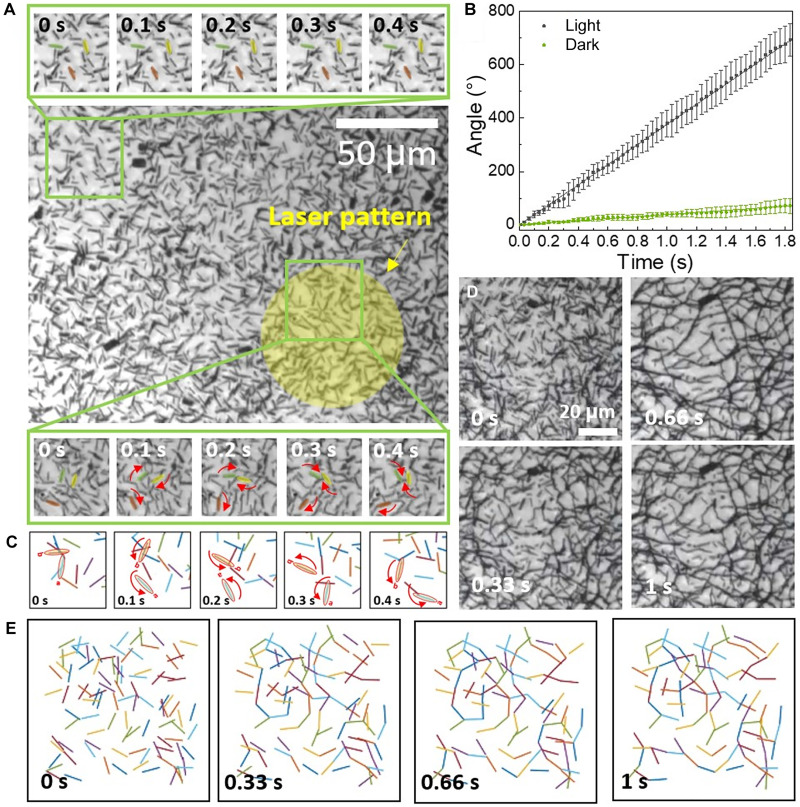
Swarm behaviors of high-density Si motor suspension in a rotating electric field under 532-nm laser illumination (100 mW/cm^2^). (**A**) At >500 kHz, motors in the light-illuminated area (highlighted in yellow) rotate with substantially increased speed (snapshots of Si motors at the bottom) compared to those in non-lit region (top). (**B**) Rotation speed analysis of Si motors with (black line) and without (green line) light illumination (60,000 V/m, 100 mW/cm^2^). (**C**) Enhanced rotation is reproduced in Brownian dynamics simulation. (**D**) Between 50 and 200 kHz, the high-density Si motors assemble into networks under light illumination; the same is observed in the snapshots of (**E**) Brownian dynamics simulation.

At an electric field frequency of 500 kHz, and under light exposure, the rotational speed of the Si motors dramatically rises from 39°/s to 377°/s, all while showing negligible near-field assembly, as illustrated in Fig. 4 (A and B) and movie S1. The position, size, and shape of the regions containing Si motors with much-enhanced rotation speed can be dynamically tuned by light, as found in the gradually expanding circle (Fig. 4A, bottom), in contrast to the non-lit region with low-speed motors (Fig. 4A, top). This collective motion is also observed in Brownian dynamic simulations. In the numerical model, high-density Si motors are dispersed in a confined region and subjected to the condition of ∣Im(α)α∣∼1, corresponding to that applied in the experiments. Although positioned closely, the motors instantly spin individually with enhanced speeds (Fig. 4C and movie S9). Although the simulation is semiquantitative with the aforediscussed simplifications, the results well reproduce the features of the swarm observed in experiments.

Between 50 and 200 kHz, the Si motors rapidly transform into 2D branched networks upon light projection (Fig. 4D and movie S10). In this frequency range, the near-field electrostatic force dominates over the electrorotation torque, given by ∣Im(α)α∣≪1. We find that, during the network formation, a Si motor at 200 kHz often rotates until it attaches to a branch of the network. The network formation is dynamic, where short and thin branches and isolated motors still rotate and occasionally disassemble and reassemble at different locations. At 100 kHz, the network stabilizes because of the much-reduced rotational torque, and the motors rapidly assemble into a giant network in the area defined by light. Meanwhile, the network also evolves with time; it begins with many branches that frame numerous microscale cells. The microcells gradually coalesce into larger cells during the merging of nearby branches. The network can instantly form and change its size, shape, and position corresponding to the light pattern and rapidly disassemble into a homogeneous suspension once the light is turned off (movie S10). Numerical simulation is conducted on the basis of the understanding of the weighted role of the near-field electrostatic attraction relative to electrorotation, given by ∣Im(α)α∣∼0. The result reproduces the network features in experiments (Fig. 4E and movie S11).

**Fig. 5.**
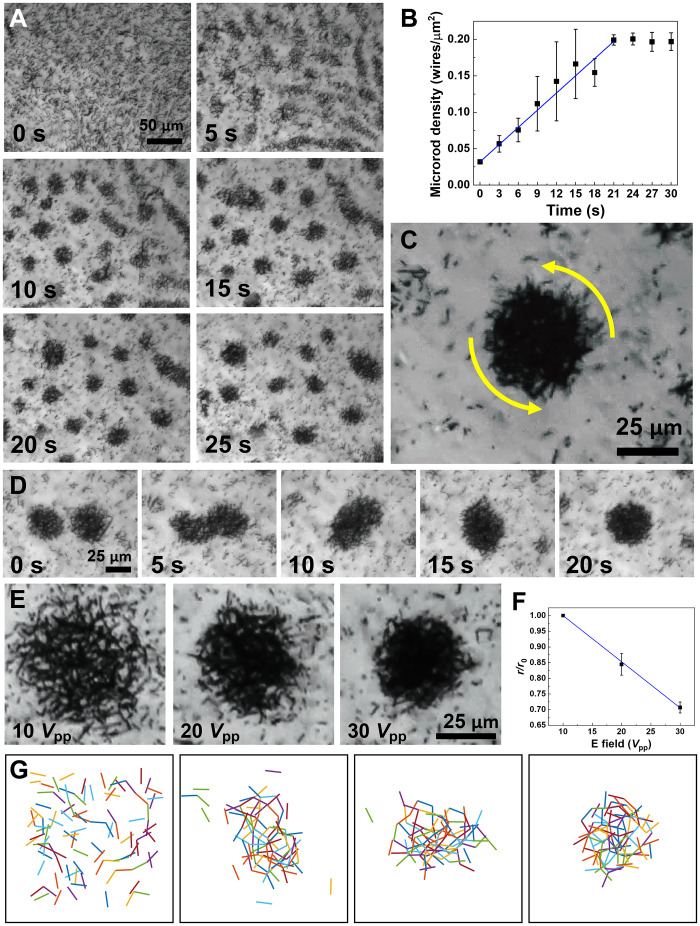
Light-directed clustering of Si micromotors. (**A**) Dynamic clustering under light illumination (532-nm laser, 100 mW/cm^2^) with snapshots taken every 5 s, and (**B**) the micromotor density changes over time. (**C**) Zoom-in image of a cluster rotating counterclockwise with the constituting micromotors spinning in the same direction and precessing along the circumference of the cluster. (**D**) Two neighboring clusters merge. (**E**) Voltage-controlled dynamic change of the size of a cluster, and (**F**) its corresponding relative radius change. (**G**) Simulation reproduces the clustering process.

Under light, at 10 kHz, both electrorotation (Fig. 3A) and electrostatic attraction (Fig. 3B) are high, and ∣Im(α)α∣∼0.6 (Fig. 3C). At such a condition, it is intriguing to examine how the Si motors interact and affect the swarming behavior. Experimentally, we find that a previously unobserved swarm mode emerges, where the Si motors rotate, gradually assemble, and nucleate into an array of clusters rotating under light (*532*-nm laser, 100 mW/cm^2^) (Fig. 5A). The clustering starts from random nucleation in a homogeneous suspension made of high-speed rotating motors, followed by gradual condensation into smaller droplet-like clusters. Here, the Si motor density linearly increases until it reaches a plateau of ~0.2 motors/μm^2^ (Fig. 5B). In a cluster, all the constituting Si motors rotate on individuals in the same direction, and some precess along the circumference of the cluster. The clustering sites are random, which could arise from stochastic local fluctuation of the density, arrangement, and thus the interaction forces of close-by micromotors. For instance, a long Si motor (or a bundle of short Si motors) can serve as a nucleation center when forming a cluster due to its higher electric polarization compared to that of an individual short Si motor. Then, nearby individually rotating micromotors are attracted to the nucleation site and gradually join the cluster. In experiments, we also find that, with the increase of the size and density of a cluster, the attraction force increases, and more rotating micromotors join the cluster. Eventually, the size of a cluster stabilizes with the depletion of micromotors near the cluster. Multiple clusters form at the same time and are distanced by the surrounding depletion regions. Each individual cluster is made of high-density micromotors stacked in several layers, all spinning along with the cluster, counter to the electric field direction (Fig. 5C and movie S3). When two clusters approach each other during spinning, they coalesce into a larger cluster, which suggests a strong mutual attraction between the clusters, as shown in Fig. 5D and movie S12. The cluster density and size can be well controlled by the applied electric voltage. As shown in Fig. 5 (E and F) and movie S13, a cluster reduces its size monotonically with the increased voltage.

With numerical simulation, we successfully reproduce the cluttering process at optimized conditions (Fig. 5G and movie S14) by implementing a high fraction of Im(α) along with a high α, corresponding to the condition of both strong rotation torque and electrostatic interaction used in experiments. With Im(α) accounting for a significant fraction of the total polarization, i.e., ∣Im(α)α∣∼1, the simulated motors can spin independently, not forming into chains as found in the frequency range of 50 to 200 kHz under 532-nm laser of 100 mW/cm^2^. In the meantime, the high total electric polarization **p** that results in electrostatic attraction prevents diffusion of the motors away from a cluster while attracting additional motors to the cluster, as observed in experiments. In our simulations, we did not attempt to fully reproduce the complex experimental observations of the system due to the intrinsic complexity of the system that involves many-body hydrodynamic, electrostatic, and electrokinetic interactions. Instead, we use a simplified model and focus on the primary parameter ∣Im(α)α∣, which dominantly governs the system's swarming behavior. By tuning this parameter, we are able to qualitatively reproduce the experimental observations. The modeling adopts a simplified approach, as hydrodynamic flow and induced electric fields generated by individual motors are not included in the simulation. In addition, at the low frequency of 10 kHz, where the clustering phenomenon is observed, electrokinetic effects such as electroosmosis and induced charge electroosmosis are strong and may also contribute to the observation.

We note that hydrodynamic flows can play roles in swarming behaviors. In our system, local hydrodynamic flow is passively generated and increases with the electrorotation. Hence, in a coarse-grain estimation, hydrodynamic flow can be considered as a complex coefficient adding to the disorderliness caused by electrorotation. Therefore, although the simulation only considers the two driving electric effects and their relative weight, the results can well reproduce the experimental observation. In addition, the agreement of the simulation and experiments highlights that the control of the weighted roles of the two countering electric effects can readily lead to the observed three swarming modes even without the assistance of hydrodynamic flow. This showcases another difference of the reported system from some magnetic swarms whose formations require hydrodynamic interactions (*35*). Experimentally, the decrease in cluster size with the increase in the electric field suggests that the countering effects of the electrorotation together with hydrodynamic repulsion have a lower weighted effect compared to the electric field attraction at a high electric field (movie S13).

### Versatile patterning and switching of swarm modes by light

**Fig. 6.**
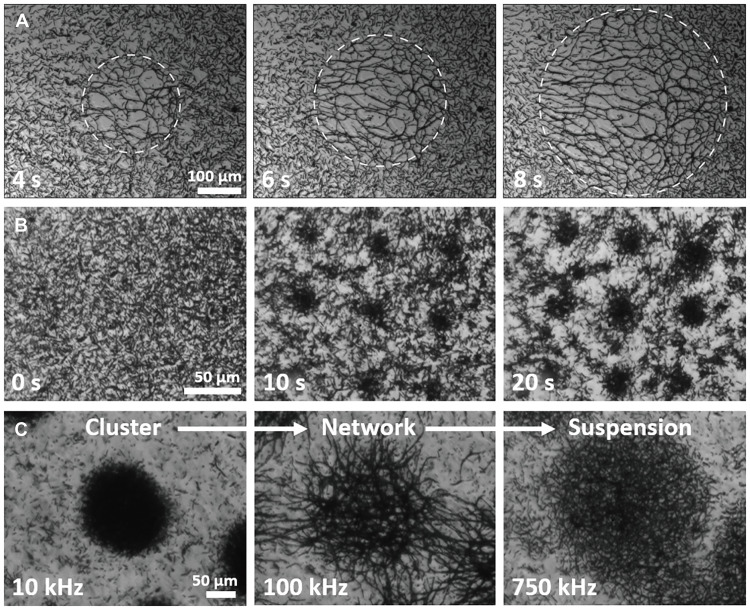
Versatile patterning and rapid, reversible switching of swarming modes by light. (**A**) Reversible dynamic network patterning under a circular light pattern with growing radii (532-nm laser, 100 mW/cm^2^, and 100 kHz, 30 *V*_pp_). (**B**) Light-patterned ordered array of rotating clusters (10 kHz). (**C**) Instant reconfiguration of droplet-like clusters to network clusters to dispersed solutions. Microelectrodes: 500-μm gap.

Last, we demonstrate the versatility in creating different swarm modes and dynamically reconfiguring their shape, size, and location with projected light patterns. As shown in Fig. 6A, under a circular light pattern with a growing diameter, a corresponding circular micromotor network instantly forms and grows from a homogeneous suspension following the light pattern at 100 kHz (movie S12). With the same method, we also assemble the motors into a rotating rectangular network (50 to 200 kHz) and transferred it to a rotating swarm with enhanced speed (500 kHz, 1 MHz) (movie S15). When a large circular pattern, e.g., 250 μm in diameter, is applied under a 10-kHz electric field, more than 10 nucleation sites emerge, which eventually coalesce into three stable clusters (fig. S3). Notably, when reducing the light pattern to a critical size, such as 50 μm in diameter, a single stable rotating cluster is formed (fig. S4). Likewise, a patterned array of clusters emerges simultaneously corresponding to the projected light pattern (fig. 6B and movie S16). Under light, a droplet cluster also instantly transforms into a network cluster, back to a droplet, disperses into solution, and reforms into a droplet in response to the AC voltage programmed at 10, 100, 750, and back to 10 kHz, respectively (Fig. 6C and movie S4).

## DISCUSSION

In summary, we report an innovative swarming-generation mechanism that can dynamically pattern micromotor swarms with unprecedented versatility, reconfigurability, and dynamic control. The constituting units, silicon micromotors, are earthly abundant, are inexpensive, and can be made in a large quantity. By exploring the interactions of visible light, Si motors, and rotating AC electric fields, multiple distinct swarming modes can be created, patterned, and switched in-between.

Three swarming modes are made, i.e., light-patterned enhanced rotation, 2D networking, and droplet clustering. The different swarming behaviors are obtained by actively tuning the relative weight of two countering interactions, the induced dipole-dipole interactions between neighboring micromotors, leading to assembly (orderliness), and the electro-rotation, leading to assembly disruption (disorderliness); both effects are offered by asynchronous rotation of Si micromotors in a rotating electric field, where electrorotation dominates in light-patterned swarms with enhanced rotation, the near-field dipole-dipole interaction governs the formation of a 2D network, and droplet clustering forms when both effects are strong. Theoretical analysis, calculation, and semiquantitative simulation well reproduce the characteristics of all collective motion, supporting our understanding of the working mechanisms. Furthermore, the multimodal swarms are dynamic and versatile, controlled by light with defined position, size, and number, which have been achieved for the first time and potentially endow micromotor swarms with previously unutilized optoelectronic and mechanical properties not offered by the constituting units (*27*, *36*).

This reported optoelectric system sets itself apart from magnetic approaches in several distinctive respects. It operates based on the interplay between light, electric fields, and semiconductor microparticles. This not only changes the driving forces but also differentiates the underlying principles that govern swarm behaviors. Specifically, our system allows fine-tuning the balance between two opposing effects: the rotational torque generated by electrorotation and the dipole-dipole attraction, from zero to one for rational control over the behaviors of the swarms.

It is because, for commonly used superparamagnetic and paramagnetic spheres, the induced dipole is always in-phase with the external magnetic field and follows the field when it rotates as the magnetization relaxation is usually much faster than the frequency of the applied external field, which is generally below the kilohertz regime due to instrumental limitations. Individual paramagnetic spheres experience no rotational torque, while chained spheres exhibit a torque that originated from the neighboring dipole-dipole interaction that tries to realign the dipoles into the external field direction, leading to synchronous rotation at low speed. However, the frequency of the applied electric field can be easily tuned across a much wider range without a loss in amplitude to tens of megahertz. This large frequency range can cover the relaxation timescale of multiple electric processes, such as ionic movement in the medium and interfacial dielectric polarization, leading to rich frequency responses. In this work, different from the counterpart magnetic rotation, the electrorotation torque is based on the imaginary part of the induced electric dipoles, which is the out-of-phase component of the electric dipoles generated by an applied electric field (rotation speed of the electric field is much higher than that of a wire, asynchronous rotation). A distinct phase difference exists between the electrorotation torque and electric dipole moment of Si nanowires at a given frequency as shown in Fig. 3 (A and B). As a result, one can readily turn on/off electrorotation and simultaneously generate high-intensity electrostatic attraction for rapidly assembling the Si wires by choosing a suitable electric field frequency and light intensity. This is predicted by the theoretical calculation in Fig. 3 (A and B) and observed in experiments.

In addition to the above mechanistic difference, equally important, the reported optoelectric swarms can be patterned in a versatile and dynamic manner, which offers the capability of spatially patterning swarm generation in the broader landscape of swarm research. Overall, this research may provide certain inspiration toward creating new swarm systems based on different actuation mechanisms and open opportunities in reconfigurable optoelectronics, active surfaces, and robotics.

## METHODS

### Fabrication of silicon nanowires

Silicon nanowires are fabricated using the MACE method with slight modifications (*37*). To briefly summarize, a monolayer of 500-nm polystyrene nanospheres is assembled on a cleaned undoped silicon wafer, followed by a deposition of a 25-nm Ag and 5-nm Au catalytic metal thin film via electron beam evaporation. The nanospheres are removed with scotch tape, resulting in a metal film with nanoholes on the silicon wafer. The wafer is then immersed in a 4.7 M hydrofluoric acid/0.3 M hydrogen peroxide etchant, dissolving the silicon underneath and resulting in arrays of nanowires. Last, silver and gold etchants are used to remove the catalytic metal layer, and the nanowires are released into DI water via sonication. The nanowires used in this work are 7 μm in average length and 500 nm in diameter, composed of undoped silicon, and stored in DI water and tested within a few days after fabrication.

### Fabrication of quadruple electrodes

Quadruple electrodes are fabricated using standard photolithography and electron-beam evaporation processes. First, a 5-nm Cr and 100-nm Au film is deposited onto a cleaned glass substrate via electron beam evaporation. The quadruple microelectrode, which consists of four rectangular pads surrounding a square area with 500 μm each side, is patterned with S1811 photoresist, designed photomasks, and commercially available Au/Cr etchants. The microelectrodes are then connected to a customized computer-controlled four-output function generator using silver-paste attached wires.

### Experimental setup

For each experiment, an ~20-μl nanowire suspension is dispersed in a polydimethylsiloxane microwell on top of the electrodes and covered by a glass slide. The nanowires gradually sink to the bottom of the well and in-plane with the electrodes. A 532-nm diode-pumped solid-state laser (Thorlabs) is used as the laser source, with a ring-shaped light-emitting diode to provide bare background light for visibility under microscope.

## Supplementary Material

20231025-1

## Supplementary Materials

### This PDF file includes:

Figs. S1 to S5 Table S1 Notes S1 to S3 Legends for movies S1 to S17
