# Supplementary material for: Light-stimulated micromotor swarms in an electric field with accurate spatial, temporal, and mode control
Source: Sci Adv. 2023 Oct 25;9(43):eadi9932. doi: 10.1126/sciadv.adi9932 (PMC10599615; doi:10.1126/sciadv.adi9932)
Supplement: Supplementary file 2 — Figs. S1 to S5 Table S1 Notes S1 to S3 Legends for movies S1 to S17 [file sciadv.adi9932_sm.pdf]

Science Advances
AAAS

Supplementary Materials for

Light-stimulated micromotor swarms in an electric field with accurate spatial, temporal, and mode control

Zexi Liang *et al.*

Corresponding author: Donglei Emma Fan, dfan@austin.utexas.edu; Zexi Liang, liangphy92@utexas.edu

Sci. Adv. **9**, eadi9932 (2023)
DOI: 10.1126/sciadv.adi9932

The PDF file includes:

Figs. S1 to S5
Table S1
Notes S1 to S3
Legends for movies S1 to S17

Other Supplementary Material for this manuscript includes the following:

Movies S1 to S17

Supplementary Information

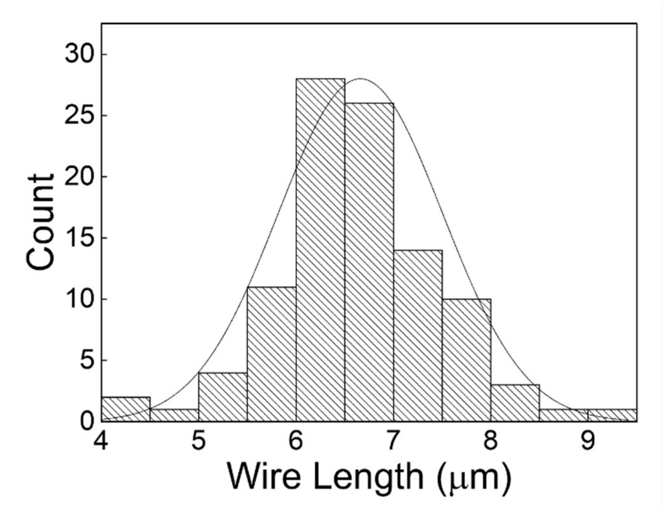

Figure S1. ***Histogram of the length of Si nanowires.*** *(measured from ~100 wires. The average is 6.7 μm)*

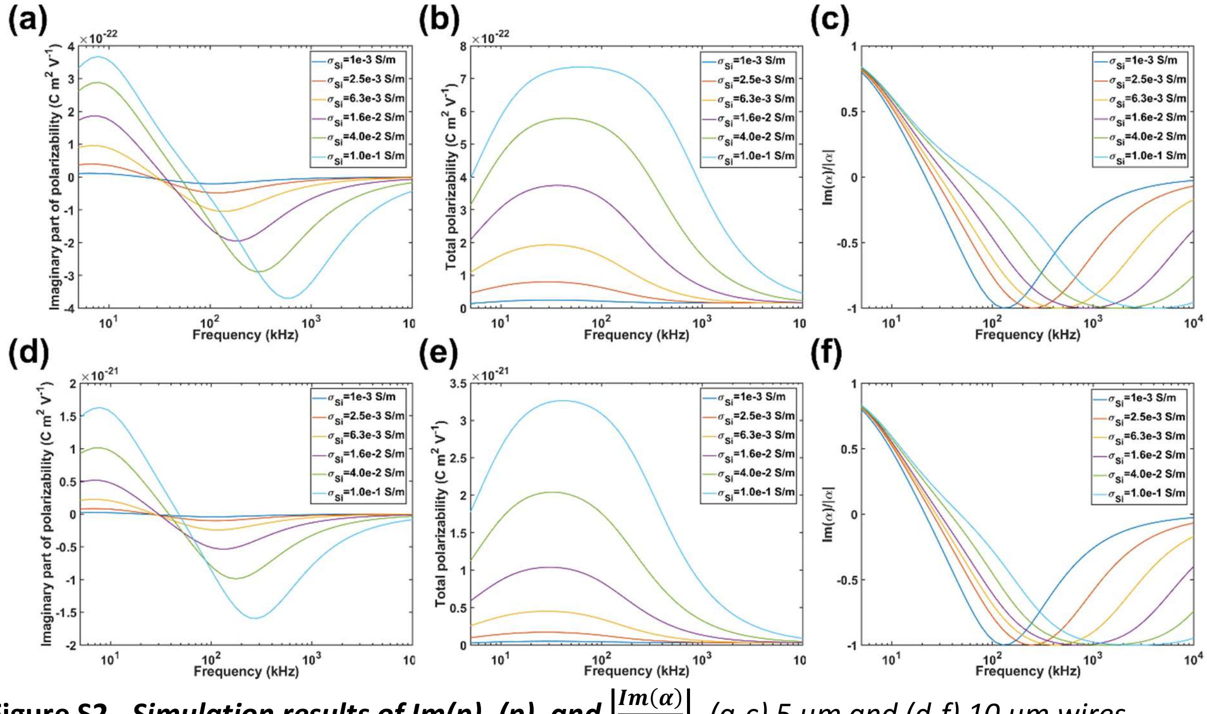

Figure S2. ***Simulation results of Im(p), (p), and*** $\left|\frac{Im(\alpha)}{\alpha}\right|$**.** *(a-c) 5 μm and (d-f) 10 μm wires.*

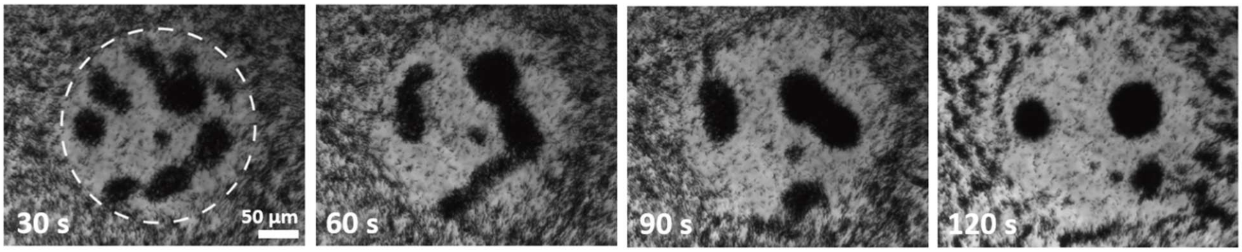

Figure S3. ***Multi-cluster formation under a large circular light pattern, e.g. 250 µm in diameter.*** *The ten small clusters eventually merge into three large stable clusters.*

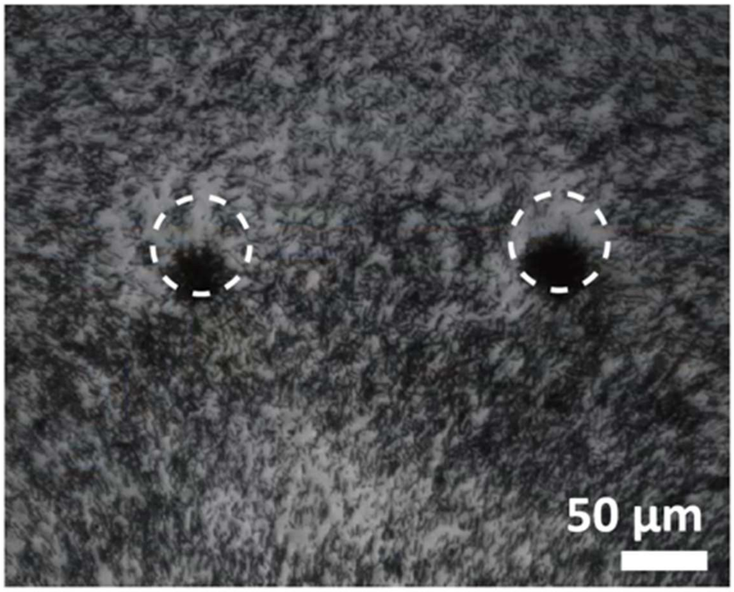

Figure S4. ***Single cluster formation when the light pattern is reduced to a size of 50 µm in diameter.***

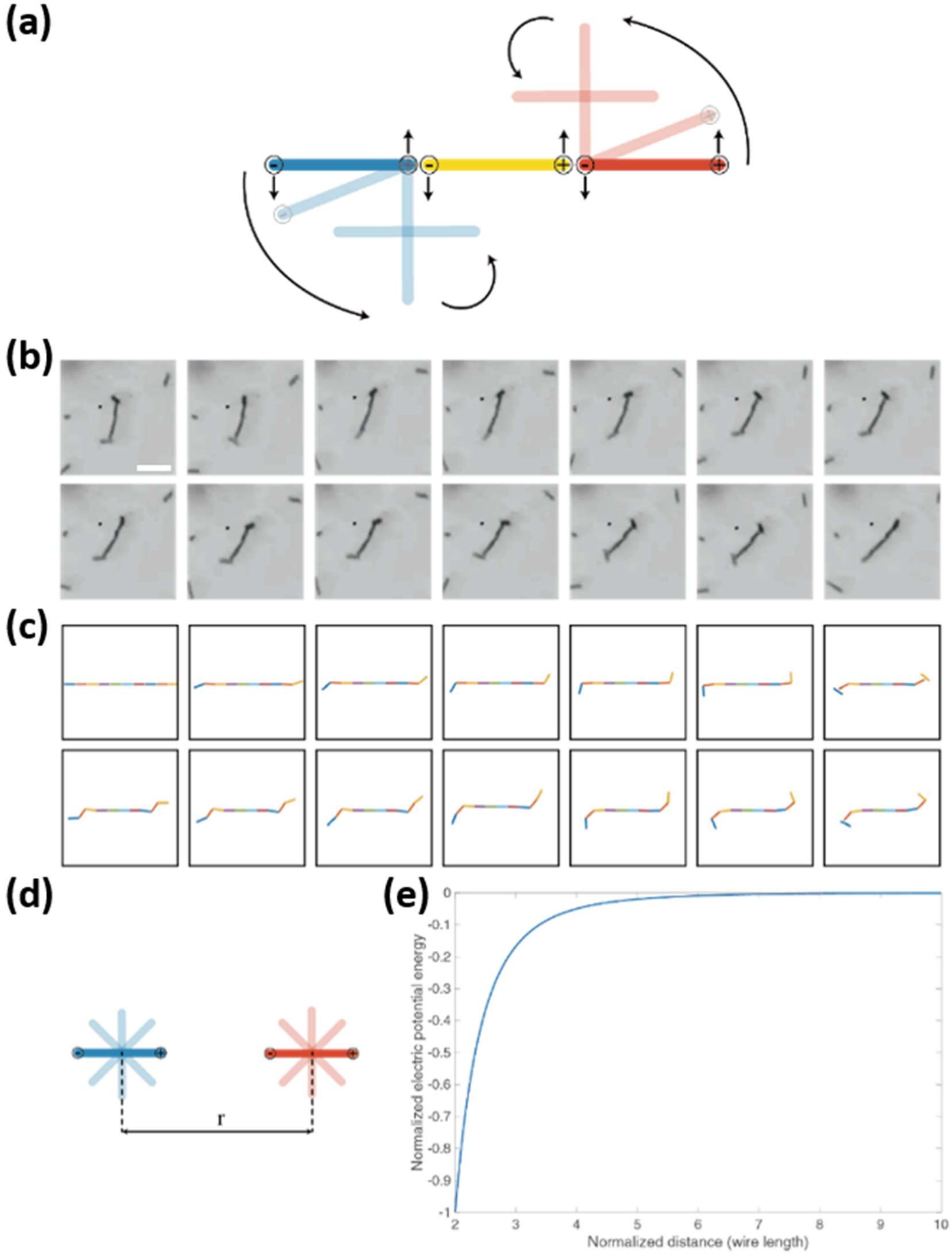

Figure S5. ***Dynamic chaining and long-range interaction of micromotor chains.*** *(a) Schematic and (b) experimental snapshots of three nanorods interacting and chaining at 200 kHz rotating E-field every 0.25 s. Scale bar: 20 μm. (c) Simulation of chaining and dynamic rotation from 10 nanorods. (d) Two polarized nanorods placed in a rotating electric field. (e) Average electric potential energy of all orientation configurations between two nanorods versus inter-distance.*

Input Stimuli	Reported Results	Concept and Working Mechanism	Reference
Acoustic	Spatial ultrasound-modulated dynamic particle assembly	Transfer of dynamic patterns: dynamically create microbubble patterns as an acoustic-field mask for generating reconfigurable nanoparticle assembly.	[3]
Acoustic	3D assembly of various materials such as silica gel particles and cells	Programmable acoustic holograms for reconfigurable 3D nanoparticle assembly	[4]
Chemical	Reversible aggregation of Au microparticles	Diffusiophoretic swarming owing to hydrazine-hydrogen peroxide reactions on Au particles; particles redisperse upon fuel consumption.	[22]
Chemical	Predator-prey interaction of active particle systems	Chemically powered mutual interactions: diffusiophoretic interactions of two different particles that power each other's motion	[23]
Electrostatic	Multimodal assembly of Janus spheres into chains, swarms, and clusters	Control of Janus spheres dipolar interactions: exploring the different dielectric responses of each hemisphere in an electric field	[9]
Magnetic	Large-scale patterning with varied degrees of order	Control of the magnetic dipole-dipole force, which is balanced by the capillary force and hydrodynamic lift force	[12]
Magnetic	Formation of Fe_3O_4 nanoparticle microswarms capable of navigation in blood vessels	Formation and controlled rolling of magnetic chains by using a rotating magnetic field with varying magnetic field strengths and frequencies.	[13]
Magnetic	Multimode transformations and locomotion of magnetic colloidal particles	Reversible collective swarm formation by using rotating and oscillating magnetic fields and their polarization.	[14]
Optical	Reversible self-assembly of various nanoparticles for chemical reaction enhancement	Control of nanoparticle water affinity *via* light-induced azobenzene ligand reactions	[5]
Optical / Chemical	UV-induced "microfireworks" motion of SiO_2-TiO_2 Janus particles	Aggregation and dispersion upon UV illumination driven by diffusiophoresis due to photocatalytic reactions on TiO_2 and UV-induced charge separation and redistribution.	[6]
Optical / Chemical	Collective "fusion" and "fission" motion of active TiO_2 particles using two different light sources	Wavelength controlled reaction switch between two hemispheres of Janus particles.	[7]
Optothermal / Chemical	Fuel-free, light-powered TiO_2 particle swarm systems with cargo-transport capabilities	TiO_2 swarm created by asymmetric ionic flow near the surface; the degree of swarm aggregation can be instantly switched by light induced O_2 generation.	[8]

Table S1. ***The input stimuli, results, concept, and driving mechanism of various micromotor swarms.***(*3*–9, *12*–*14*, *22, 23*)

Note 1: Theoretical analysis of electric polarizability of a nanorod in a rotating electric field

Consider the x-axis along a nanorod's longitudinal direction, the field vector can be expressed as $\boldsymbol{E} = \boldsymbol{E_0}\,(\hat{\mathbf{x}} - \mathrm{i}\hat{\mathbf{y}})\exp(i\omega t)$, rotating counterclockwise at angular frequency of ω. Assuming all the nanorods in the solution have identical geometry and electrical properties, the calculated polarizability (α) would be also the same. As a nanorod has a large aspect ratio, its electric polarizability along the transverse direction can be considered as negligible in the tested frequencies, and α can be approximated as the electric polarizability along the long direction of the nanorod. In an AC electric field, due to the Maxwell-Wagner polarization and electrical-double-layer effect,(*33*) the polarizability α is a function of frequency and also a complex quantity containing a phase lag to the applied external field. The induced dipole from an individual nanorod is $\boldsymbol{p} = \alpha \boldsymbol{E}_{\parallel}$. Since the field is constantly rotating, the phase of the field is the angle of the field vector. A uniform electric field always exerts a zero dielectrophoretic force on the induced dipole, but still applies a torque when the dipole moment has lagging angle to the phase of $\boldsymbol{E}_{\parallel}$. The averaged torque over one cycle can be expressed by $\boldsymbol{\tau_e} = \frac{1}{2}\mathrm{Re}\left[\underline{\mathbf{p}}(\mathbf{t}) \times \underline{\mathbf{E}}^*(\boldsymbol{t})\right] = -\frac{1}{2}E_0^2\,\mathrm{Im}[\alpha]\hat{\mathbf{z}}$. Here, as mentioned in the main manuscript, the underlines of *p(t)* and *E(t)* denote complex variables with phasor.

Note 2: Method for the calculation of electrostatic interactions of two identical nanorods in a rotating electric field

The orientation of the nanorod is defined in the interval of $(-\pi/2, \pi/2]$. The component of the electric field $\boldsymbol{E}(t)$ that is in parallel with nanorods 1 and 2 are $\boldsymbol{E}_{\parallel\mathbf{1}} = E_0\exp(i\omega t - i\theta_1)\,\hat{\boldsymbol{s}}_i$ and

$\boldsymbol{E}_{\parallel 2} = E_0 \exp(i\omega t - i\theta_2)\,\hat{\boldsymbol{s}}_j$, respectively, with correspondingly induced dipoles of $\boldsymbol{p_1} = \alpha \boldsymbol{E}_{\parallel 1}$ and $\boldsymbol{p_2} = \alpha \boldsymbol{E}_{\parallel 2}$. Since there is a phase lag of $(\theta_2 - \theta_1)$ between $\boldsymbol{E}_{\parallel 1}$ and $\boldsymbol{E}_{\parallel 2}$, the same phase lag will pass to the induced dipole $\boldsymbol{p_1}$ and $\boldsymbol{p_2}$. The dipole on nanorod 1 is then represented by two point-charges $q_1^+ = \frac{|\boldsymbol{p}_1|}{l}$ and $q_1^- = -q_1^+$ located at $\boldsymbol{r_1^+} = \boldsymbol{r}_1 + \frac{l}{2}\hat{\boldsymbol{s}}_1$ and $\boldsymbol{r_1^-} = \boldsymbol{r}_1 - \frac{l}{2}\hat{\boldsymbol{s}}_1$. The dipole-dipole interaction between the nanorods 1 and 2 is then calculated from the Coulomb forces between the four point-charges $q_1^+, q_2^+, q_1^-\ and\ q_2^-$. Since the nanorods are identical,$|q_1^+| = |q_2^+| = |q_1^-| = |q_2^-|$, when they are at the same condition. The induced charges are directly proportional to the dipole moment, and thus the same phase lag between $\boldsymbol{p_1}$ and $\boldsymbol{p_2}$ will be inherited by the charges of $q_1^\pm = \pm q_0 \exp(i\omega t - i\theta_1)\ and\ q_2^\pm = \pm q_0 \exp(i\omega t - i\theta_2)$, respectively, where $q_0 = \frac{\alpha E_0}{l}$ when a rod aligns with the external field (E). As a result, the Coulomb's force between $q_1^\pm$ and $q_2^\pm$ in average over one period is given by Equation (1):

$$F_{12} = \frac{\omega}{2\pi}\int_0^{\frac{2\pi}{\omega}} \frac{1}{4\pi\varepsilon_m}\frac{\mathrm{Re}(q_1^\pm)\mathrm{Re}(q_2^\pm)}{\left|\boldsymbol{r_1^\pm} - \boldsymbol{r_2^\pm}\right|^2}\mathrm{d}t = \frac{\cos(\theta_2 - \theta_1)}{2}\frac{1}{4\pi\varepsilon_m}\frac{q_0^2}{\left|\boldsymbol{r_1^\pm} - \boldsymbol{r_2^\pm}\right|^2}, \quad (1)$$

where ε_m is the permittivity of the suspension medium. The equation shows that all the involved point-charge interaction becomes zero in a rotating electric field when two nanorods are oriented orthogonally, corresponding to $|\theta_1 - \theta_2| = 0$.

Note3: Discussion about long-range interaction and transition between networks and clusters
The two types of swarm behaviors, network formation and clustering, only arise when the induced dipole-dipole interaction is significant, *i.e.*, under laser exposure and when the frequency is between 10 kHz to 200 kHz. But how does the transition between these two modes occur and what makes the difference, *e.g.*, how can the network form without further collapsing into clusters? We investigate the underlying principle by modeling the dynamic assembling process of a micromotor chain. As shown in **Fig. S5a**, when three nanorods assemble into a chain, the rod in the middle (in yellow) cannot receive an effective rotational torque exerted by the external electric field due to the attachment of the two neighboring nanorods at its two tips, where the opposite charges at the junctions nullify electric torques from the external field. For the two micromotors separated at the two ends the chain, however, has one end fixed to the rod in the middle, with the other end free to rotate. As a result, the two nanorods on the ends of the chain (in blue and red) rotate around the fixed tips of the nanorod in the center. When they are approximately 90° to the adjacent rod (in yellow), the dipole-dipole interaction becomes much lower, often causing them to disassemble from the chain (**Fig. S5b**). This experimental result is well replicated in the simulation (**Fig. S5c** and **Movie S17**), where two micromotors rotate around the tips of a close-by nanorod assembled in the center, frequently disassemble when aligned to the orthogonal direction, and re-assemble at the tips during the rotation.

The simulation of the above multi-nanorod chain system sheds light to the two aforediscussed swarm modes. When many nanorods are dispersed in a suspension, the high density and near-field interaction result in their rapid assembly with neighboring rods, forming into a network in an electric field. For the nanorods assembled inside the network, the rotational torques are

largely cancelled, similar to those in the center of the chain system studied in the simulation (**Fig. S5c**). Here, although long-range attraction forces among the rods can exist at frequencies of 10 kHz to 200 kHz, such electrostatic interactions are insufficient to overcome the energy barrier needed to break the assembled nanorod chain for the clusters observed at 10 kHz. On the other hand, at 10 kHz, the electrorotation torque is much more substantial compared to that of ~200 kHz, which greatly suppresses chaining or assembly, while the electrostatic interaction is strong enough that can aggregate the rotating nanorods into clusters. To unveil the electrostatic attraction effect that could account for cluster formation, we calculate the electrostatic potential between two polarized nanorods placed in a rotating electric field in **Fig. S5d**. As aforediscussed in Equation (2), the dipole-dipole electric interaction between two neighboring rods can be either attractive or repulsive depending on their relative orientations. When rotating the two rods, it is reasonable to assume that their relative orientation is uniformly distributed in the range of $(-\pi/2, \pi/2]$. With this assumption, we can calculate the average electrical potential between two randomly oriented rods under the rotating electric field as a function of the center-to-center distance, as shown in **Fig. S5e**, which indicates the averaged electrostatic force between two rotating nanorods in an electric field is attractive.

Supplementary Movies

Movie S1: Enhanced rotation of Si micromotors under an expanding circular light pattern at 500 kHz
Movie S2: Network formation of Si micromotors under a circular light pattern (100 kHz)
Movie S3: Cluster formation of Si micromotors under light at 10 kHz
Movie S4: Instant transformation between modes *via* frequency change
Movie S5: Assembly of two neighboring Si micromotors at 100 kHz
Movie S6: Light-controlled switching of assembly/independent spinning of two Si micromotors at 50 kHz
Movie S7: Simulation of two individually spinning Si micromotors
Movie S8: Simulation of two Si micromotors that assemble and rotate as a single entity
Movie S9: Simulation of a group of Si micromotors with enhanced rotation
Movie S10: Network formation of Si micromotors at 100 kHz
Movie S11: Simulation of network formation of a group of Si micromotors
Movie S12: Merging of two Si micromotor clusters (10 kHz)
Movie S13: Control of cluster density and size *via* E-field modulation
Movie S14: Simulation of clustering of a group of Si micromotors
Movie S15: Dynamically rotating Si micromotor swarms under a rotating rectangular light pattern (50 kHz, 100 kHz, 200 kHz, 500 kHz, 1 MHz)
Movie S16: Cluster formation of Si micromotors under a light pattern (10 kHz)
Movie S17: Simulation of chaining and dynamic rotation of 10 micromotors
